# Mechanistic Insights into Tyrosinase-Catalyzed Metabolism of Hydroquinone: Implications for the Etiology of Exogenous Ochronosis and Cytotoxicity to Melanocytes

**DOI:** 10.3390/ijms262110734

**Published:** 2025-11-04

**Authors:** Shosuke Ito, Ludger Kolbe, Tamara Rogers, Tobias Mann, Gudrun Weets, Hitomi Tanaka, Tomoko Nishimaki-Mogami, Thierry Passeron, Makoto Ojika, Kazumasa Wakamatsu

**Affiliations:** 1 Institute for Melanin Chemistry, Fujita Health University, Toyoake 470-1192, Japan; hitanaka@u-gifu-ms.ac.jp (H.T.); kwaka@fujita-hu.ac.jp (K.W.); 2 Research and Development, Beiersdorf AG, 20245 Hamburg, Germany; ludger.kolbe@beiersdorf.com (L.K.); tamara.rogers@beiersdorf.com (T.R.); tobias.mann@beiersdorf.com (T.M.); gudrun.weets@beiersdorf.com (G.W.); 3 Department of Medical Technology, School of Health Sciences, Gifu University of Medical Science, Seki 511-3892, Japan; 4 Department of Biochemistry, National Institute for Health Sciences, Kawasaki-ku, Kawasaki 210-1501, Japan; mogami@nihs.go.jp; 5 INSERM U1065, Centre de Médecine Moléculaire, University Côte d’Azur, 06200 Nice, France; 6 Department of Applied Biosciences, Graduate School of Bioagricultural Sciences, Nagoya University, Nagoya 464-8601, Japan; ojika@agr.nagoya-u.ac.jp

**Keywords:** hydroquinone, benzoquinone, 2-hydroxyhydroquinone, 2-hydroxybenzoquinone, eumelanin, pheomelanin, 4-aminophenol, exogeneous ochronosis, tyrosinase

## Abstract

The metabolism of hydroquinone (HQ) by tyrosinase presents significant biochemical and dermatological challenges, particularly due to its association with adverse effects such as exogenous ochronosis (EO). Despite its widespread use in skin-lightening products, the detailed mechanistic pathways of HQ metabolism by tyrosinase remain inadequately understood. This study aims to elucidate the mechanistic insights into the tyrosinase-catalyzed metabolism of HQ, leading to the production of HQ-eumelanin (HQ-EM) and HQ-pheomelanin (HQ-PM). We employed HPLC analysis to detect key intermediates and final metabolites. Results show that mushroom tyrosinase catalyzes the hydroxylation of HQ to 2-hydroxyhydroquinone (HHQ) via the 2-hydroxybenzoquinone (HBQ) pathway, giving rise to HQ-EM. However, in the presence of cysteine, a shift from HBQ to the benzoquinone (BQ) pathway occurs, giving rise to HQ-PM. Hydroiodic acid hydrolysis of HQ-PM and subsequent HPLC-electrochemical analysis identified 4-aminophenol (AP) as degradation product, thereby serving as a novel marker to monitor HQ oxidation in vitro. These results indicate that HQ functions both as a “pseudo” substrate for tyrosinase—undergoing redox exchange with dopaquinone to form BQ—and as a true substrate, yielding HBQ. This dual role contributes to the formation of HQ-EM and HQ-PM. It would be possible that EO is caused by a continuous oxidation of HQ mediated by tyrosinase activity in the skin.

## 1. Introduction

Hydroquinone (HQ) is an aromatic organic compound that has gained widespread recognition for its extensive use in both pharmaceutical and cosmetic applications. Structurally characterized by two hydroxyl groups attached to a benzene ring with a *para*-substitution, HQ exhibits significant chemical reactivity, making it a versatile agent suitable for a variety of applications. In the 1930s, HQ was discovered as a depigmenting agent [1,2] and since then, HQ was considered the gold standard for treatment of hyperpigmentation disorders such as melasma, lentigines, and post-inflammatory hyperpigmentation [3,4,5,6]. Despite the widespread use of HQ in dermatology, the mechanistic pathways of its metabolism remain incompletely understood.

Tyrosinase, a multifunctional enzyme, catalyzes the hydroxylation of monophenols to *o*-diphenols and the subsequent oxidation of *o*-diphenols to *o*-quinones [7,8]. This enzymatic activity is central to melanin biosynthesis by converting tyrosine to the melanin precursor dopaquinone [9]. However, due to the similarity between HQ and melanogenic precursors, HQ can interfere with this pathway, suppressing melanin production and resulting in skin lightening over time. Additionally, HQ-induced generation of reactive oxygen species (ROS) and quinones leads to the oxidative damage of membrane lipids and proteins such as tyrosinase, further contributing to the lightening effect [10].

Despite its therapeutic value, HQ has been associated with notable adverse effects, particularly when used in high concentrations or over extended periods [11,12,13,14,15]. One of the most significant complications is exogenous ochronosis (EO), a skin condition characterized by bluish-black hyperpigmented patches in the treated areas. The reactive species derived from HQ are thought to induce polymerization of proteins in the dermis, potentially leading to the formation of those ochronotic particles found in EO [11,13]. Involvement of tyrosinase has been suggested as a possible cause of EO [16]; however, solid evidence has not been presented.

**Figure 1:**
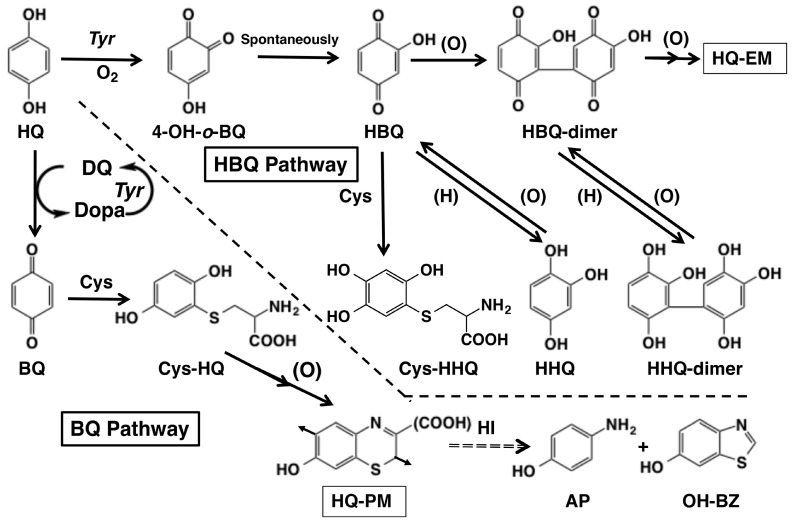
Possible metabolic pathways of hydroquinone (HQ) in the tyrosinase-catalyzed oxidation. Direct action of tyrosinase on HQ yields 4-hydroxy-1,2-benzoquinone (4-OH-*o*-BQ) which spontaneously isomerizes to 2-hydroxy-1,4-benzoquinone (HBQ). When L-cysteine (Cys) is not present, HBQ gradually dimerizes to form HBQ-dimer which gradually forms hydroquinone-eumelanin (HQ-EM) or HBQ-oligomer. In the presence of Cys, HBQ reacts rapidly to form 5-*S*-cysteinyl-2-hydroxy-1,4-hydroquinone (Cys-HHQ). These reactions are named the HBQ pathway. Indirect action of tyrosinase on HQ through participation of dopaquinone (DQ) yields 1,4-benzoquinone (BQ). When Cys is present, BQ reacts rapidly to form 2-*S*-cysteinyl-1,4-hydroquinone (Cys-HQ). Cys-HQ is oxidized to form hydroquinone-pheomelanin (HQ-PM). These reactions are named the BQ pathway. Hydroiodic acid (HI) hydrolysis of HQ-PM yields 4-aminophenol (AP) and 6-hydroxybenzothiazole (OH-BZ) as markers. The arrows in the structure of HQ-PM indicate the point of connection to the adjacent monomer units.

In a recent study [17], we have shown that HQ, acting as “pseudo” substrate for tyrosinase, is oxidized by human tyrosinase in the presence of L-dopa, a natural substrate, suggesting a possible role of HQ in the etiology of EO. That study gave significant insights into the metabolic processes involving HQ in human melanocytes, specifically identifying tyrosinase as a key enzyme in the oxidation of HQ. In the present study, we aimed to further elucidate the mechanistic insights into the tyrosinase-catalyzed metabolism of HQ, especially at the later stages leading to the production of polymeric melanin-like pigments, hydroquinone-eumelanin (HQ-EM), and hydroquinone-pheomelanin (HQ-PM; Figure 1). We used commercially available mushroom tyrosinase in place of human tyrosinase. The use of human tyrosinase limited additional examinations, because (1) the enzyme is strongly inhibited by L-cysteine [18,19] and (2) the tyrosinase preparation contains a high concentration of imidazole [17]. Furthermore, since the main HQ pathways analyzed in this study are downstream of tyrosinase and proceed non-enzymatically, they are independent of the initial tyrosinase enzyme activity, be it from human or mushroom tyrosinase. We employed high-performance liquid chromatography-UV detection (HPLC-UVD) to detect and identify the formation of key intermediates and final metabolites during tyrosinase action on HQ. Two metabolic pathways are possible for tyrosinase-catalyzed oxidation of HQ: the 2-hydroxybenzoquinone (HBQ) pathway through the direct hydroxylation of HQ [20] and the benzoquinone (BQ) pathway through redox exchange with dopaquinone (DQ; Figure 1) [21,22].

## 2. Results

### 2.1. Oxidation of HQ by Mushroom Tyrosinase

**Figure 2:**
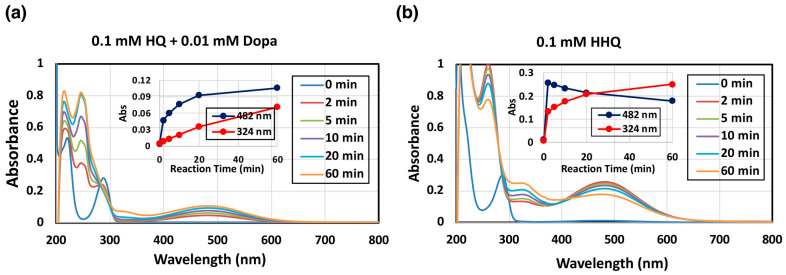
Spectrophotometric following mushroom tyrosinase-catalyzed oxidation of HQ and HHQ at pH 7.4 and at 37 °C. (**a**) 0.1 mM HQ in the presence of 0.01 mM L-dopa and (**b**) 0.1 mM HHQ. The experiments were conducted twice, and good reproducibility was obtained. The figure was from a single experiment and representative.

Oxidation of HQ was investigated to determine if a possible phenolic substrate would be oxidized by mushroom tyrosinase to produce the corresponding *o*-quinone [23]. HQ itself was not oxidized by mushroom tyrosinase without the presence of L-dopa. However, when a catalytic amount (0.1 mol eq.) of L-dopa was added as a co-substrate, HQ was gradually oxidized by mushroom tyrosinase, resulting in the formation of a red chromophore with absorption maximum at 482 nm (Figure 2a). The 482 nm pigment was identified as HBQ since mushroom tyrosinase oxidized 2-hydroxyhydroquinone (HHQ) to form a chromophore with an identical absorption (Figure 2b). Additionally, another chromophore with an absorption at 324 nm was observed (Figure 2b), which may correspond to HBQ-dimer (see below for the identification).

**Figure 3:**
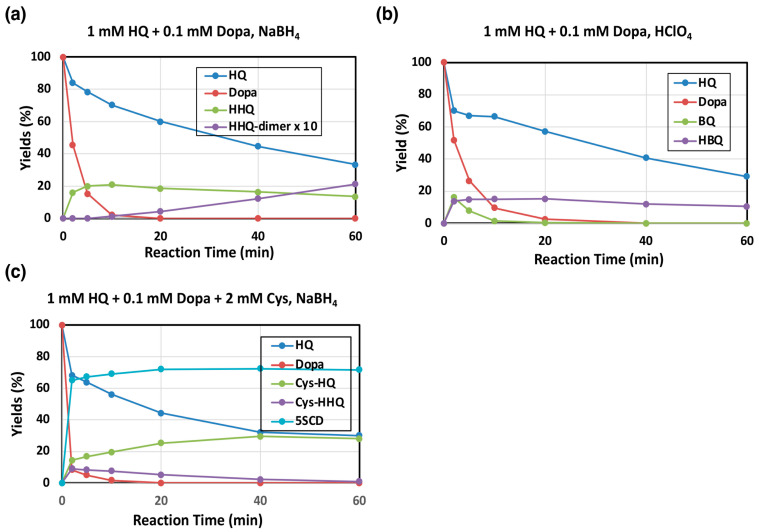
Time course of mushroom tyrosinase-catalyzed oxidation of HQ. HPLC analysis following the oxidation at pH 7.4. (**a**,**b**) 1 mM HQ in the presence of 0.1 mM L-dopa and (**c**) 1 mM HQ in the presence of 0.1 mM L-dopa and 2 mM L-cysteine (Cys). (**a**,**c**) The reaction was stopped by the addition of NaBH_4_. (**b**) The reaction was stopped by HClO_4_. (**b**) HBQ standard is not available because of its instability. Therefore, the yield of HBQ was determined by assuming that the combined yield of HQ, BQ, and TBQ was 100% at 2 min. Data were obtained for two independent experiments with good reproducibility and the average of both is shown. Note that yields of HHQ-dimer are those for monomer units (×2 of dimers).

To examine the reaction pathway more precisely, we followed the tyrosinase-catalyzed oxidation of 1 mM HQ in the presence of 0.1 mM L-dopa at pH 7.4, and products were analyzed by HPLC-UVD in the form of reduced, polyphenolic compounds obtained by reduction with NaBH_4_. We usually use 0.1 mM concentration of substrate to follow its conversion. However, to minimize the effect of auto-oxidation, we used 1 mM substrate concentration here. HQ was gradually oxidized to form HBQ (analyzed as HHQ) (Figure 3a). HHQ, in its oxidized form HBQ, was found to slowly decay, resulting in the accumulation of HHQ-dimer as identified via NMR analysis by direct comparison with a synthetic HHQ-dimer. It was previously reported that BQ was also produced during this oxidation [22]. To confirm the production of BQ, the oxidation was terminated with HClO_4_ (with no NaBH_4_ reduction). As shown in Figure 3b, BQ was in fact produced, but it rapidly disappeared due to continuing reactions. The BQ pathway appears to proceed faster than the HBQ pathway when we take the high reactivity of BQ into account.

The effect of Cys on tyrosinase oxidation of HQ was then examined because Cys participates in the production of PM due to its rapid reaction with quinones [9,23]. As shown in Figure 3c, Cys effectively participated in the reaction, yielding 2-*S*-cysteinylhydroquinone (Cys-HQ) in 38% yield at 60 min as identified by the addition reaction of Cys to BQ. The Cys adduct to HBQ, 5-*S*-cysteinyl-2-hydroxyhydroquinone (Cys-HHQ), was also produced but in lower yields (Figure 1), whereas HHQ was not detected. It is important to note that Cys-HQ was produced in a yield approximately 1.6-fold greater than Cys-HHQ (at 2 min, 15.5% and 9.5%, respectively), indicating that the production of BQ proceeds faster than the production of HBQ, suggesting that the BQ pathway predominates over the HBQ pathway. In this reaction, L-dopa acts as a substrate of tyrosinase to form DQ, leading to two competitive reactions: the redox reaction to produce BQ and the addition reaction to produce 5-*S*-cysteinyldopa (5SCD) [9].

### 2.2. Oxidation of HHQ by Mushroom Tyrosinase

**Figure 4:**
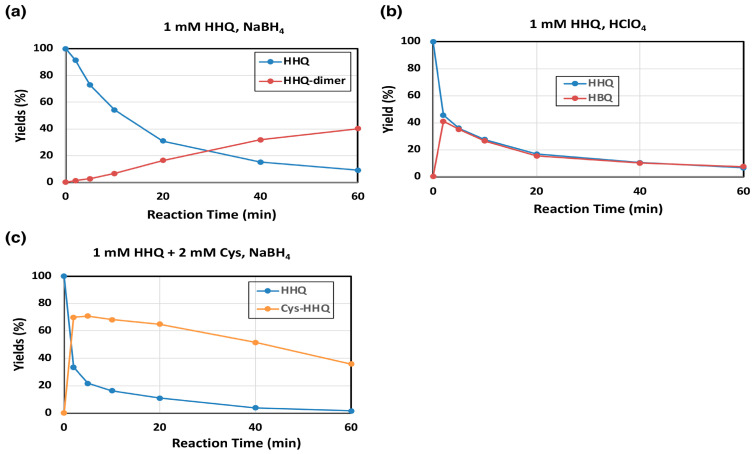
Time course of mushroom tyrosinase-catalyzed oxidation of HHQ. HPLC analysis following the oxidation at pH 7.4. (**a**) 1 mM HHQ and (**b**) 1 mM HHQ in the presence of 2 mM Cys. (**a**,**c**) The reaction was stopped by the addition of NaBH_4_. (**b**) The reaction was stopped by HClO_4_. Data were obtained for two independent experiments with good reproducibility and the average of both is shown. Note that yields of HHQ-dimer are those for monomer units (×2 of dimers).

HHQ is known to act as a substrate of mushroom tyrosinase [20]. Oxidation of HHQ by mushroom tyrosinase was examined to know how the immediate product HBQ is metabolized at the next stage. Consistent with the spectral observation (Figure 2b), HHQ is in fact a good substrate of mushroom tyrosinase and produced HBQ, HHQ-dimer (Figure 4a,b) or Cys-HHQ in the presence of 2 mol eq. Cys, respectively (Figure 4c). The time course of HHQ oxidation by mushroom tyrosinase is presented in Figure 4a. HBQ (analyzed as HHQ after NaBH_4_ reduction) was immediately formed and decayed gradually to produce HHQ-dimer (40% yield at 60 min). To confirm the oxidation of HHQ to HBQ, the reaction was terminated with HClO_4_ (with no NaBH_4_ reduction). As shown in Figure 4b, HBQ was in fact produced, but it gradually decayed due to production of HBQ-dimer (identified as HHQ-dimer in Figure 4a). It is noted that the oxidation of HHQ by tyrosinase has slowed down after 10 min (Figure 4a,b). This may be ascribed to the suicide inactivation of tyrosinase that occurs during the tyrosinase-catalyzed oxidation of HHQ [24]. Another interesting point is that the decay curves of HHQ and HBQ overlap to each other, suggesting that HBQ reacts with HHQ to form HBQ-dimer. HHQ acts as a nucleophile, while HBQ acts as an electrophile.

A standard of HHQ-dimer was prepared by tyrosinase oxidation of HHQ, and the structure was confirmed by ^1^H- and ^13^C-NMR (Supplemental Figure S1 and Table S1) and high-resolution MS. The ^1^H-NMR analysis of the HHQ-dimer exhibited the presence of *o*-substituted and *p*-substituted hydrogen atoms, which indicates the structure 2, 2’, 3, 4’, 5’, 6-hexahydroxybiphenyl (Supplemental Figure S1 and Table S1). The ^13^C-NMR spectrum is also consistent with this structure (Supplemental Figure S1 and Table S1). The high-resolution MS spectrum gave a major peak, consistent with [M-3H]^-^ ion. The UV absorption spectrum of HHQ-dimer showed an absorption maximum at 294 nm (in 0.1 M HCl), compared to a maximum of HHQ at 288 nm. The production of HHQ-dimer has been postulated by MS analysis on the product of nitrate oxidation of HHQ, but evidence was not sufficient to confirm the structure [25].

Tyrosinase-catalyzed oxidation of HHQ in the presence of Cys was then examined. As shown in Figure 4c, Cys rapidly reacted with the immediate and transient product HBQ to form a Cys adduct, Cys-HHQ. This HQ derivative was recently identified as a metabolite of HQ by human tyrosinase [17]. Cys-HQ was not produced in the reaction, confirming that Cys-HQ can only be produced from BQ but not from HBQ.

### 2.3. Production of HQ-EM and HQ-PM and Finding of a Marker, 4-Aminophenol, Derived from HQ-PM

We then attempted to find marker compounds that serve to monitor HQ oxidation in vitro and in vivo, if feasible. This concept stemmed from the fact that dopa-derived EM and PM provide various useful markers upon degradation reactions like alkaline hydrogen peroxide oxidation (AHPO) [26,27] and HI reductive hydrolysis [26,28]. AHPO of dopa-EM affords pyrrole-2,3,5-tricarboxylic acid (PTCA) and pyrrole-2,3-dicarboxylic acid (PDCA), while AHPO of dopa-PM yields thiazole-2,4,5-tricarboxylic acid (TTCA) and thiazole-2,3-dicarboxylic acid (TDCA) in addition to PTCA and PDCA [26,27].

**Figure 5:**
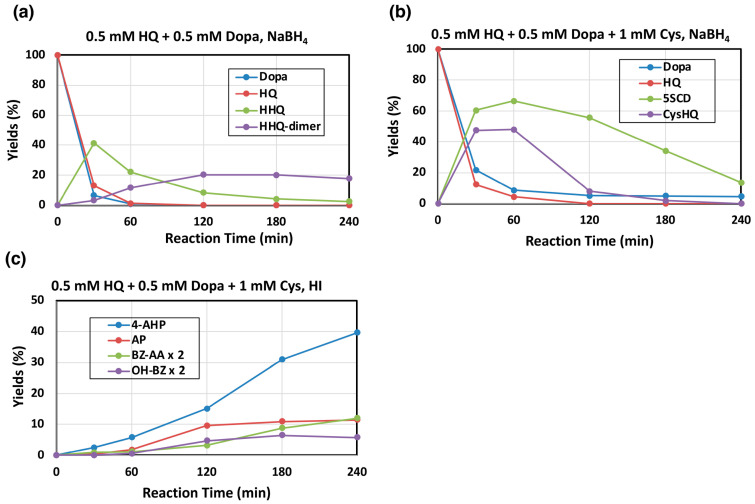
Time course of the mushroom tyrosinase-catalyzed oxidation of a mixture of HQ, L-dopa, and Cys. HPLC analysis following the oxidation at pH 7.4. (**a**) 0.5 mM HQ and 0.5 mM L-dopa, (**b**) 0.5 mM HQ, 0.5 mM L-dopa, and 1 mM L-cysteine (Cys). (**c**) The oxidation mixtures in (**b**) were analyzed for PM markers. (**a**,**b**) For the determination of the reaction products, the reaction was stopped by the addition of NaBH_4_, and (**c**) for HI hydrolysis, the reaction was stopped by the addition of 1 M HCl. Data were obtained for two independent experiments with good reproducibility and the average of both is shown. BZ-AA and OH-BZ were analyzed by HPLC-UVD at 300 nm. Note that yields of HHQ-dimer are those for monomer units (×2 of dimer).

A solution of EM from HQ and L-dopa was prepared through co-oxidation of a mixture of 0.5 mM HQ and 0.5 mM L-dopa by mushroom tyrosinase for 240 min. This longer reaction time was selected to follow the later stages of oxidation. Most of HQ and L-dopa were rapidly oxidized within 30 min (Figure 5a) and HQ was converted to HBQ (identified as HHQ), followed by gradual oxidization to HBQ-dimer (identified as HHQ-dimer). The oxidation mixtures were subjected to AHPO; however, no useful markers deriving from HQ-EM were afforded. Only PTCA and PDCA were detected as major products arising from dopa-EM.

We next examined mushroom tyrosinase-catalyzed oxidation of 0.5 mM HQ and 0.5 mM L-dopa in the presence of 1 mM Cys as a model of pheomelanogenesis incorporating HQ and L-dopa. As shown in Figure 5b, both HQ and L-dopa were mostly consumed within 30–60 min and the Cys addition products of BQ and DQ (Cys-HQ and 5-*S*-cysteinyldopa, respectively) were produced in good yields. Cys-HQ then rapidly disappeared due to oxidation to form HQ-PM while 5-*S*-cysteinyldopa disappeared more slowly to form dopa-PM. Subsequent HI hydrolysis and HPLC-electrochemical detection of the oxidation mixtures identified 4-amino-3-hydroxyphenylalanine (4-AHP; 40%) arising from dopa-PM [28,29] but 4-aminophenol (AP; 11%), that can derive from Cys-HQ via benzothiazine compounds (Figure 1), was also identified. AP was recently found to serve as a marker of HQ-PM produced in cells expressing human tyrosinase [17]. Furthermore, HPLC-UVD of the HI hydrolysates detected not only the dopa-PM marker 6-alanyl-4-hydroxybenzothiazole (benzothiazole amino acid, BZ-AA; 6.0% at 240 min) [29], but also 6-hydroxybenzothiazole (OH-BZ; 3.3% at 180 min) (Figure 5c). These results demonstrate that AP (and possibly OH-BZ) can be used as a newly discovered marker of HQ-PM.

Comparison of Figure 5a,b shows that HHQ, a major product of HBQ pathway, was produced in a 41% yield at 30 min, while Cys-HQ, a major product of the BQ pathway in the presence of cysteine, was produced in 47% yield. This result suggests that a shift from the HBQ pathway to the BQ pathway occurs when cysteine is present.

## 3. Discussion

Two pathways are possible for tyrosinase-catalyzed oxidation of HQ: the HBQ pathway and the BQ pathway (Figure 1). In a recent study using human tyrosinase [17], we demonstrated that both pathways proceed with the BQ pathway being a major one. However, the presence of imidazole in the enzyme preparation made the direct identification of the product BQ impossible. Also, the inhibition of tyrosinase activity by L-cysteine strongly suppressed the reaction. Our present study demonstrated that mushroom tyrosinase catalyzes hydroxylation of HQ to produce a hydroxylated product HHQ in its oxidized form HBQ (the HBQ pathway, Figure 1). HBQ is formed through tautomerization of 4-hydroxy-1,2-benzoquinone (4-OH-*o*-BQ) that is initially produced, through the phenol oxidase activity of *oxy-*tyrosinase (Figure 1) [8,30]. To convert *met*-tyrosinase, a major form of native tyrosinase, to *oxy*-tyrosinase through *deoxy*-tyrosinase, catechols or other reductants such as ascorbic acid are required [20]. In melanocytes, L-dopa may act to produce *oxy*-tyrosinase. HBQ, once formed, gradually decays to form an HBQ-dimer. The HBQ-dimer is also unstable, giving rise to the production of HQ-EM. This pathway of tyrosinase-catalyzed metabolism of HQ is compatible with the selective melanocyte toxicity of HQ through a possible production of ROS. In this regard, Penney et al. [31] suggested that the toxic mechanism proceeds via the oxidation of HQ to toxic compounds in melanocytes.

The production of HHQ during oxidation of HQ by mushroom tyrosinase has been previously documented [20,21,22,30]. It has also been reported that in the absence of L-dopa, HQ is much less effective as a substrate compared to L-tyrosine, a natural substrate for tyrosinase [21]. Importantly, the present study is the first to isolate and characterize the underivatized HHQ-dimer.

Another metabolic pathway for HQ is the BQ pathway (Figure 1). This pathway involves the redox exchange reaction between DQ, an immediate product of *met*-tyrosinase oxidation of L-dopa, and HQ to form dopa and BQ. This pathway has been proposed by Palumbo et al. [21,22] for tyrosinase oxidation of HQ. The present study unambiguously identified this pathway not only by direct comparison through HPLC but also by trapping BQ with Cys to form Cys-HQ. In the presence of a thiol like Cys, oxidation of HQ resulted in the production of Cys-HQ rather than Cys-HHQ, demonstrating a shift from the HBQ pathway to the BQ pathway. Cys-HQ can then undergo further oxidation to form a pheomelanic pigment. In our recent study [16], we have shown that HQ acts as a “pseudo” substrate for human tyrosinase through the redox oxidation of HQ to form BQ by DQ. In the present study, this metabolic pathway was clearly confirmed.

Since dopa-derived EM and PM provide various useful markers upon degradation reactions, we attempted to find HQ-PM-specific markers. HI hydrolysis of HQ-PM gives AP just like dopa-PM gives AHP isomers [29]. In our recent study [17], analyses of HQ-exposed melanocytes and tyrosinase-transfected cells confirmed the production of AP after HI hydrolysis [17], indicating that the oxidation of HQ to BQ did in fact take place in melanocytes. Production of Cys-HQ (and glutathionyl-HQ) was also confirmed in the tyrosinase-transfected T 293 T cells [17]. It should be noted, however, that in those in vitro experiments, we faced difficulties due to cytotoxicity and metabolites production that are independent of tyrosinase activity. This may be ascribed to the tendency of HQ to undergo auto-oxidation to form BQ and subsequent oligomeric products.

## 4. Materials and Methods

### 4.1. Materials

L-Dopa, L-cysteine (Cys), hydroquinone (HQ), 4-aminophenol (AP), and mushroom tyrosinase (2687 U/mg) were purchased from Sigma-Aldrich (St. Louis, MO, USA). 2-Hydroxy-1,4-hydroquinone (HHQ, 1,2,4-trihydroxybenzene) and 6-hydroxybenzothiazole (OH-BZ) were from Tokyo Chemical Industry (Tokyo, Japan). 1,4-Benzoquinone (BQ) was from FUJI FILM Wako Pure Chemical (Osaka, Japan). 6-(2-Amino-2-carboxyethyl)-4-hydroxybenzothiazole (benzothiazole amino acid, BZ-AA) was prepared as described in Wakamatsu et al. [29]. The preparation of 2-hydroxyhydroquinone-dimer (HHQ-dimer) is described below.

### 4.2. Analytical Methods

An HPLC system was used to follow the course of tyrosinase oxidation and consisted of a JASCO 880-PU pump (JASCO Co.), an Osaka Soda C18 column (Capcell Pak MG; 4.6 × 250 mm; 5 μm particle size; Osaka Soda, Osaka, Japan), and a JASCO UV/VIS detector (JASCO Co., Tokyo, Japan) at 290 nm (for HQ, L-dopa, HHQ, HHQ-dimer, Cys-HQ, and Cys-HHQ, and 5SCD) or 250 nm (for BQ and HBQ) or a Shiseido electrochemical detector (Tokyo, Japan) at 500 mV versus a Ag/AgCl electrode (4-AHP and AP), unless otherwise described. The mobile phase was 0.4 M formic acid: methanol, 95:5 or 90:10 (*w*/*w*). For the analysis of BQ and HBQ, it was necessary to elute them fast with 0.4 M formic acid: methanol, 70:30 (*w*/*w*), to avoid adsorption onto the column matrix. Analyses were performed at 45 °C and at a flow rate of 0.7 or 1.0 mL/min. For preparative separation of HHQ-dimer, an Osaka Soda C18 preparative column (Capcell Pak MG; 20 × 250 mm; 5 μm particle size) was used at 45 °C and at a flow rate of 7.0 mL/min with the same mobile phase used for the analytical separation.

UV/visible spectra were analyzed with a JASCO V-600 UV-VIS spectrophotometer (JASCO Co.). ^1^H NMR (400 MHz) and ^13^C NMR (100 MHz) spectra were obtained in 0.1 M DCl using a Bruker AVANCE 400 spectrometer (Billerica, MA, USA). Chemical shifts were referenced to the solvent (formic acid) signals (8.23 ppm for ^1^H and 168.4 ppm for ^13^C). High-resolution mass spectra were obtained using a 6220 TOF mass spectrophotometer (mode: electrospray ionization—time-of-flight, negative; ESI(−)-TOF) (Agilent Technologies, Santa Clara, CA, USA).

### 4.3. Tyrosinase-Catalyzed Oxidation of HQ or HHQ

A solution (2 mL) of 1 mM HQ or HHQ was oxidized by tyrosinase (250 U/mL) at 37 °C in 50 mM sodium phosphate buffer (pH 7.4) in the presence of L-dopa and L-cysteine (Cys) when necessary. Aliquots of the reaction mixtures were periodically taken up and subjected to HPLC analysis. For reductive termination of the reaction, 200 μL aliquots were mixed with 20 μL 10% NaBH_4_, followed by 180 μL 0.8 M HClO_4_. To analyze BQ or HBQ, 200 μL aliquots were mixed with 200 μL 0.8 M HClO_4_ to keep the quinones not reduced (Figure 3b and Figure 4b).

### 4.4. Preparation of HHQ-Dimer

A solution of 252 mg (2 mmol) HHQ in 50 mM sodium phosphate buffer, pH 6.8 (196 mL) was oxidized by 50,000 U mushroom tyrosinase (in the buffer, 4 mL) at 25 °C. The mixture was vigorously stirred for 60 min. Then, the dark wine-red solution was treated with 200 mg NaBH_4_ to give a yellow solution, to which 5 mL 6 M HCl was added. The mixture was extracted with ethyl acetate (100 mL, twice) and the combined extracts washed with 40 mL sat. NaCl soln, and dried over Na_2_SO_4_. Evaporation of the ethyl acetate extract and preparative HPLC (0.4 M formic acid: methanol = 95:5) followed by lyophilization gave 16.9 mg (6.8% yield) of HHQ-dimer as a pale brown powder. ^1^H-NMR and ^13^C-NMR (Supplemental Figure S1 and Table S1). HHQ-dimer, *m*/*z* 247 ([M−3H]^−^) as a base peak. High-resolution MS 247.0256, calcd for C_12_H_7_O_6_, 247.0242. UV (in 0.1 M HCl) λ_max_ 294 nm (ε 3400 per monomer).

## 5. Conclusions

In conclusion, our study shows that mushroom tyrosinase can use HQ as a substrate, leading to the production of polymeric melanin-like HQ derivatives. Depending on the presence or absence of Cys, oxidation of HQ by mushroom tyrosinase eventually results in the formation of either HQ-PM or HQ-EM via their corresponding BQ or HBQ pathways.

Possible implications of this study are briefly discussed. Both the HBQ and the BQ pathways may lead to cytotoxicity to melanocytes [17]. HBQ and its oligomers are likely to produce ROS through redox reactions characteristic of their polyphenol structure [20], while BQ is highly reactive with proteins via conjugation through cysteinyl residues, thus leading to its cytotoxicity [31,32,33,34]. It is well known that BQ and its derivatives are highly cytotoxic [35] and genotoxic [36]. Additionally, HQ-PM may act as a pro-oxidant, like dopa-PM [37], to exert melanocyte toxicity. It may be emphasized that the production of oxidative stress has been shown to play an important role in melanoma pathogenesis [38].

Another clinically relevant point is the involvement of HQ in inducing EO. EO is a severe adverse effect seen in some users of hydroquinone-containing products after long-term use [11,12,13,14,15]. The etiology of EO had been an enigma for a long time. A prior hypothesis suggested that the development of EO might be linked to the inhibition of homogentisate dioxygenase by HQ due to the resemblance of EO and endogenous ochronosis in patients with alkaptonuria [39,40]. However, this hypothesis was proved to be unlikely in our recent study [17]. Rather, it may be possible that some low-molecular HQ metabolites such as BQ, HBQ, HBQ-dimers, Cys-HQ, and Cys-HHQ penetrate the dermis, bind to dermal fibers, and start the polymerization of ochronotic particles to induce EO [17].

Importantly, the demonstration that HQ serves as a substrate in both the BQ and HBQ pathways challenges the long-standing notion of HQ as a tyrosinase inhibitor. This finding implies that the depigmenting effect of HQ is unlikely to result from direct inhibition of tyrosinase but rather involves an alternative mechanism such as oxidative damage to melanocytes which results in reduced production of melanin. In relation to this, it should be added that HQ is considered the criterion standard for the treatment of skin hyperpigmentation, although its mechanism of depigmenting effects is still unclear [41]. New generation of cosmetic depigmenting agents with well identified mechanisms of action, at least efficient as 4% HQ, and with better tolerance should be preferred for treating melanin hyperpigmentary disoders [41]. Further research is required to elucidate the precise mechanism underlying HQ’s skin-lightening properties.

Finally, as a technical note, it should be cautioned that the production of HQ-EM might interfere with some viability assays such as MTT assay because the HBQ chromophore of HQ-EM possesses a strong absorption around 480 nm.

## Data Availability

Data are contained within the article and Supplementary Materials.

## Supplementary Materials

The following supporting information can be downloaded at: https://www.mdpi.com/article/10.3390/ijms262110734/s1.

## Abbreviations

The following abbreviations are used in this manuscript:

HQ	Hydroquinone
EO	Exogenous ochronosis
EM	Eumelanin
PM	Pheomelanin
HHQ	2-Hydroxy-1,4-hydroquinone
HBQ	2-Hydroxy-1,4-benzoquinone
BQ	1,4-Benzoquinone
AP	4-Aminophenol
ROS	Reactive oxygen species
Cys	L-Cysteine
HPLC-UVD	High-performance liquid chromatography-UV detection
DQ	Dopaquinone
4-OH-*o*-BQ	4-Hydroxy-1,2-benzoquinone
HQ-EM	Hydroquinone-eumelanin
Cys-HHQ	5-*S*-Cysteinyl-2-hydroxy-1,4-hydroquinone
Cys-HQ	2-*S*-Cysteinyl-1,4-hydroquinone
HQ-PM	Hydroquinone-pheomelanin
HI	Hydroiodic acid
OH-BZ	6-Hydroxybenzothiazole
5SCD	5-*S*-Cysteinyldopa
AHPO	Alkaline hydrogen peroxide oxidation
PTCA	Pyrrole-2,3,5-tricarboxylic acid
PDCA	Pyrrole-2,3-dicarboxylic acid
TTCA	Thiazole-2,4,5-tricarboxylic acid
TDCA	Thiazole-2,3-dicarboxylic acid
4-AHP	4-Amino-3-hydroxyphenylalanine
BZ-AA	6-(2-Amino-2-carboxyethyl)-4-hydroxybenzothiazole
