# Supplementary material for: Mechanistic Insights into Tyrosinase-Catalyzed Metabolism of Hydroquinone: Implications for the Etiology of Exogenous Ochronosis and Cytotoxicity to Melanocytes
Source: Int J Mol Sci. 2025 Nov 4;26(21):10734. doi: 10.3390/ijms262110734 (PMC12610078; doi:10.3390/ijms262110734)
Supplement: Supplementary file 1 [file ijms-26-10734-s001.zip › ijms-3941832-supplementary.pdf]

Supplementary Materials

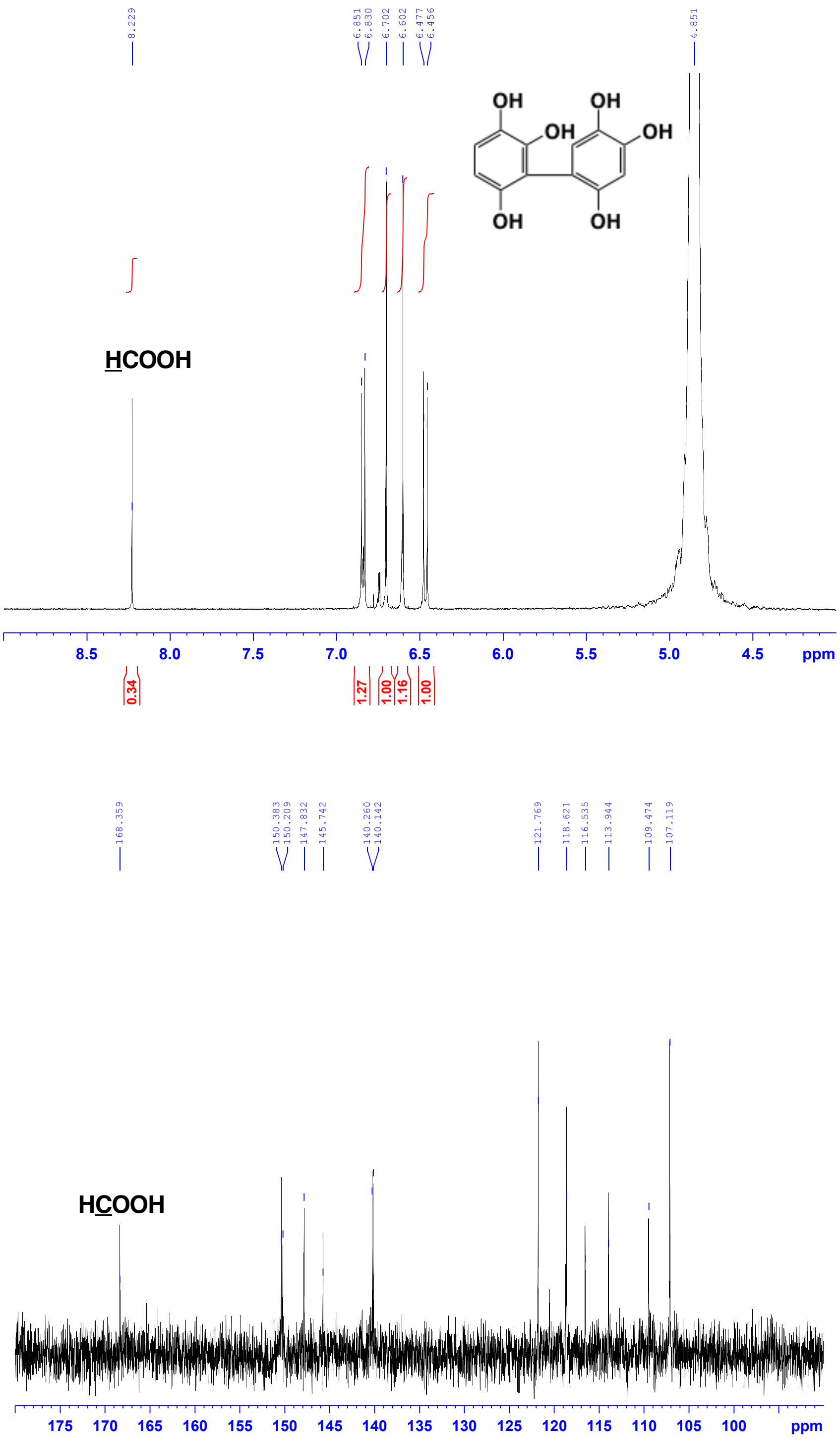

Supplementary Figure S1. NMR spectra of HHQ-dimer. (top) ^{1}H-NMR, (bottom) ^{13}C-NMR. Note that the signals at 8.23 ppm in ^{1}H-NMR and 168.4 ppm in ^{13}C-NMR are due to the residual HCOOH. Taken in 0.1 M DCl.

Supplementary Table S1. Assignments of signals from ^{1}H and ^{13}C NMR spectra (in DCl) of HHQ-dimer.

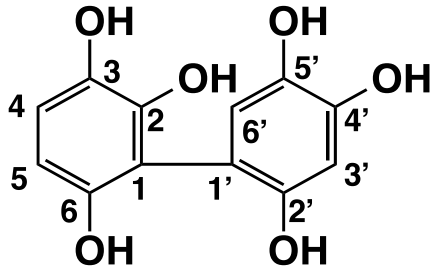

Position	HHQ-dimer		
	δ_H	Mult., *J* (Hz)	δ_C
1			118.6
2			147.8
3			140.1[a]
4	6.84	d, 8.4	113.9
5	6.47	d, 8.4	109.5
6			150.2[b]
1’			121.8
2’			150.4[b]
3’	6.60	S	107.1
4’			145.7
5’			140.3[a]
6’	6.70	S	116.5

a, b: interchangeable within the same sign.
